# Pulsed radiotherapy to mitigate high tumor burden and generate immune memory

**DOI:** 10.3389/fimmu.2022.984318

**Published:** 2022-10-06

**Authors:** Duygu Sezen, Hampartsoum B. Barsoumian, Kewen He, Yun Hu, Qi Wang, Chike O. Abana, Nahum Puebla-Osorio, Ethan Y. Hsu, Mark Wasley, Fatemeh Masrorpour, Jing Wang, Maria Angelica Cortez, James W. Welsh

**Affiliations:** ^1^ Department of Radiation Oncology, The University of Texas MD Anderson Cancer Center, Houston, TX, United States; ^2^ Department of Radiation Oncology, Koç University School of Medicine, Istanbul, Turkey; ^3^ Department of Radiation Oncology, Shandong Cancer Hospital and Institute, Shandong First Medical University and Shandong Academy of Medical Sciences, Jinan, China; ^4^ Department of Bioinformatics and Computational Biology, the University of Texas MD Anderson Cancer Center, Houston, TX, United States

**Keywords:** radiotherapy, immunotherapy, pulsed radiation, lung cancer, immune memory

## Abstract

Radiation therapy (XRT) has a well-established role in cancer treatment. Given the encouraging results on immunostimulatory effects, radiation has been increasingly used with immune-check-point inhibitors in metastatic disease, especially when immunotherapy fails due to tumor immune evasion. We hypothesized that using high-dose stereotactic radiation in cycles (pulses) would increase T-cell priming and repertoire with each pulse and build immune memory in an incremental manner. To prove this hypothesis, we studied the combination of anti-CTLA-4 and Pulsed radiation therapy in our 344SQ non-small cell lung adenocarcinoma murine model. Primary and secondary tumors were bilaterally implanted in 129Sv/Ev mice. In the Pulsed XRT group, both primary and secondary tumors received 12Gyx2 radiation one week apart, and blood was collected seven days afterwards for TCR repertoire analysis. As for the delayed-Pulse group, primary tumors received 12Gyx2, and after a window of two weeks, the secondary tumors received 12Gyx2. Blood was collected seven days after the second cycle of radiation. The immunotherapy backbone for both groups was anti-CTLA-4 antibody to help with priming. Treatment with Pulsed XRT + anti-CTLA-4 led to significantly improved survival and resulted in a delayed tumor growth, where we observed enhanced antitumor efficacy at primary tumor sites beyond XRT + anti-CTLA-4 treatment group. More importantly, Pulsed XRT treatment led to increased CD4^+^ effector memory compared to single-cycle XRT. Pulsed XRT demonstrated superior efficacy to XRT in driving antitumor effects that were largely dependent on CD4^+^ T cells and partially dependent on CD8^+^ T cells. These results suggest that combinatorial strategies targeting multiple points of tumor immune evasion may lead to a robust and sustained antitumor response.

## Introduction

Radiation therapy is one of the critical components of cancer treatment, especially in local advanced and metastatic settings. Despite encouraging results with XRT and other local or conventional systemic strategies, disease progression is still an issue that needs further research.

The advantage of radiation was previously thought to arise entirely from reducing tumor burden and accordingly improved local control. However, further understandings regarding the role of the immune system in cancer has led to the recognition that radiation therapy is a critical component for immunogenic cell death. Radiation provokes the release of neoantigens and primes the immune system to attack cancer cells outside the radiation field. Therefore, it functions as an in-situ vaccine and augments immune-mediated tumor regression. Preclinical studies have demonstrated that radiation can expand T-cell priming and tumor-specific antigen presentation (1, 2).

Over the last decade, novel therapeutic approaches that drive the immune system to identify and attack cancer cells have caused a paradigm shift in the oncologic era. These therapies usually provide durable responses to multiple solid and hematologic malignancies. Immunotherapy is now a cornerstone of cancer patients’ treatment in this context.

In recent years, there has been a growing interest in using XRT and immunotherapy together as part of oncologic management. Radiation may be significant in treating immunologically ‘cold’ tumors that usually do not have an adequate response to immunotherapy (3, 4). In addition, combining XRT with immunotherapy is reasonable, considering that radiation works in synergy to enhance systemic control and improve outcomes for tumors that acquire resistance to immunotherapy after a certain duration of treatment. KEYNOTE-001 was a multicenter, phase 1 trial in which anti-PD-1 antibody pembrolizumab was evaluated in progressive locally advanced or metastatic NSCLC patients (5). In the secondary analysis of KEYNOTE-001, patients were divided into subgroups to compare those who previously received radiotherapy with patients who had not (6). In this analysis, the patients who underwent previous radiotherapy reported having more prolonged progression-free survival and overall survival with pembrolizumab treatment than patients who did not have previous radiotherapy.

The abscopal effect was described in 1953 (7) and refers to systemic (out of the radiotherapy field) antitumoral effects caused by local radiotherapy. While it was previously known as a rare phenomenon, a growing body of preclinical and clinical data supports that the abscopal effect is immune meditated; therefore, radiation and immunotherapy combinations potentiate abscopal effects (2, 8–10). In a pooled analysis of two randomised trials, pembrolizumab alone and pembrolizumab plus radiotherapy were compared in metastatic non-small cell lung cancer (11). The best abscopal response rate was 19.7% with pembrolizumab versus 41.7% with pembrolizumab plus radiotherapy (p = 0.0039), and the best abscopal disease control rate was 43.4% with pembrolizumab while it was 65.3% with pembrolizumab plus radiotherapy (p = 0.0071). Median progression-free survival (4.4 months vs. 9 months; p = 0.045) and median overall survival (8.7 months vs. 19.2 months; p = 0.0004) was also significantly longer in the combined arm.

Recent data strongly suggest that using radiation to treat as many sites as possible effectively potentiates the systemic effects of both radiation and immunotherapy (12–14). Therefore, RT in cycles may be especially advantageous for polymetastatic disease as an effective and safe way to irradiate more metastatic lesions without hindering clinical workload.

Although synergistic effects of radiation and immunotherapy have been demonstrated in preclinical models, additional studies have yet to address factors, such as the most effective administration of therapies, to overcome low response rates and acquired resistance (15). Recently, an ablative radiation dosing scheme as pulses of ten days apart was evaluated in a Mouse model and it was reported that radiation therapy dosing and scheduling is critical for tumor control, in combination with checkpoint blockade therapies (16).

The concept of ‘Pulsed-XRT’ was based on the hypothesis that using high-dose stereotactic radiation in cycles (pulses) would increase T-cell priming and repertoire with each pulse and incrementally build immune memory with or without immunotherapy (17).

## Materials and methods

### Cell lines and drugs

The 344SQ parental lung adenocarcinoma cell line (344SQ-P) was a generous gift from Dr. Jonathan Kurie at MD Anderson Cancer Center. All experiments involved either 344SQ parental (344SQP) lung adenocarcinoma cell line, or previously derived 344SQ anti-PD1 resistant (344SQR) cell line (18). For the current study, 344SQP and 344SQR cells were cultured in RPMI-1640 media supplemented with 10% fetal bovine serum and penicillin/streptomycin. Mouse anti-CTLA-4 IgG2b (BioXcell) was diluted in phosphate-buffered saline (PBS; pH 7.4).

### Mice

The experimental mice were 129Sv/Ev syngeneic females aged 12-16 weeks. Mice were purchased from Taconic Biosciences and bred in-house at the Experimental Radiation Oncology animal facility at the University of Texas MD Anderson Cancer Center according to the Animal Care IACUC guidelines.

### Tumor inoculation and treatment

**Figure 1:**
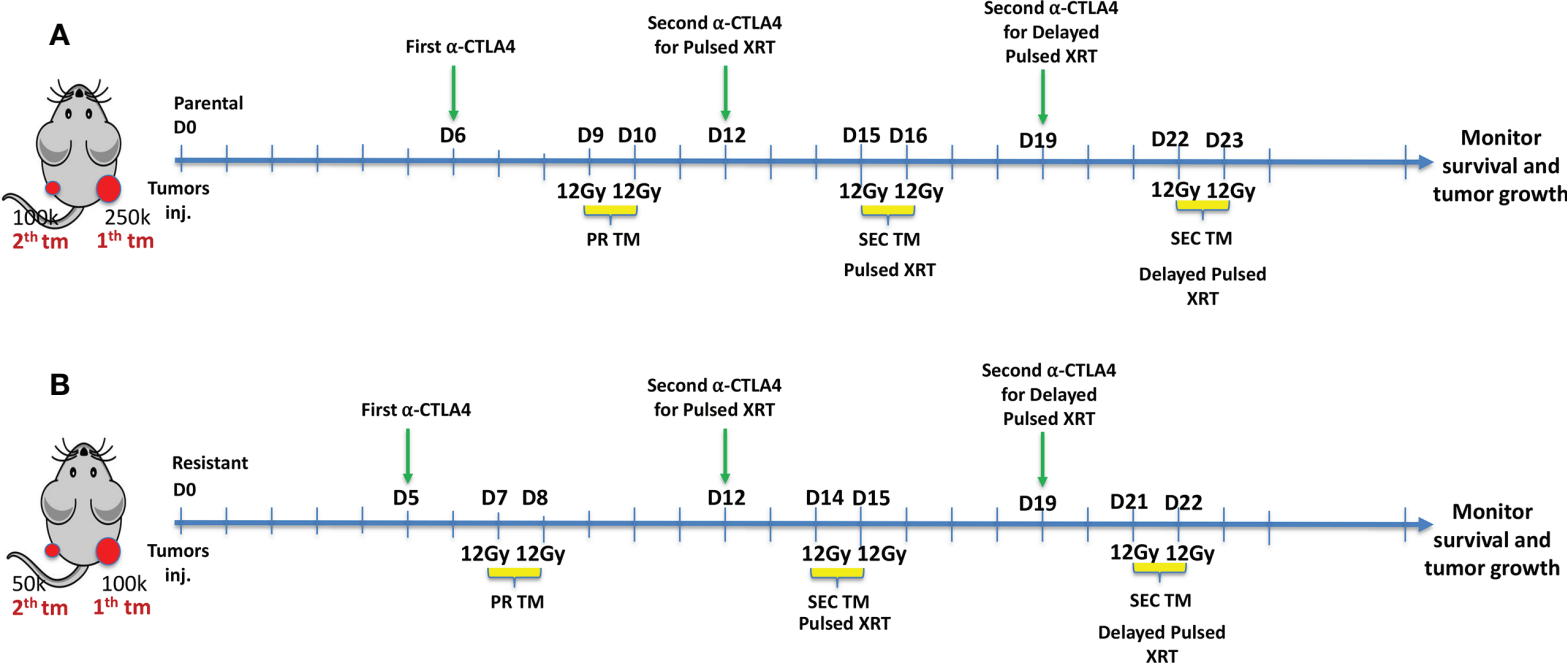
**(A)** Experimental design for Pulsed XRT (12Gy given on days 9, 10, 15, 16) and Delayed Pulsed XRT (12Gy given on days 9, 10, 22, 23) in 344SQ-Parental murine model. **(B)** Experimental design for Pulsed XRT (12Gy given on days 7, 8, 14, 15) and Delayed Pulsed XRT (12Gy given on days 7, 8, 21, 22) in 344SQ-Resistant model.

A murine model of lung adenocarcinoma was developed to demonstrate the efficacy of Pulsed XRT. To establish primary tumors, 2.5x10^5^ 344SQP cells were injected subcutaneously (s.c.) into the right hind legs of 12 to 16-weeks-old female 129Sv/Ev mice. On the same day, 1x10^5^ 344SQP cells were injected s.c. into the left hind leg as secondary tumors (**Figure 1A**). For the resistant cell line, cell numbers were adjusted to 1x10^5^ for the primary and 0.5x10^5^ for the secondary tumors (**Figure 1B**).

In XRT only and XRT+anti-CTLA-4 groups, radiation (12Gyx2) was given to the right hind leg on days 9 and 10 for the 344SQP model and days 7-8 for the 344SQR model. In addition to radiation to the primary tumors, mice in the Pulsed XRT and Pulsed XRT+anti-CTLA-4 groups receieved radiation to the secondary tumors with the same dose as the primary tumor on days 15 and 16 for the 344SQP model, and on days 14 and 15 for the 344SQR model. Mice in the Delayed Pulsed dose radiation group receeived radiation to the left hind leg on days 22-23 for 344SQP and 21-22 for 344SQR.

Anti-CTLA-4 was given intraperitoneally (i.p) as two shots (50 μg per shot) on either day 5 or 6 and day 12. For the mice in the delayed Pulsed XRT group, the second dose of anti-CTLA-4 treatment was administered on day 19 to provide the same time interval between the second injection and the second cycle of radiation with the other groups. The timeline of these treatments is shown graphically in **Figures 1A, B**. Primary and secondary tumors were measured twice per week using digital calipers, and volumes were calculated using the length × width^2^/2 formula.

### Analysis of lung metastases

Lungs were harvested from tumor-bearing mice when mice were euthanized because of tumor volume. They were stored in Bouin’s fixative solution (Polysciences, Warrington, PA; Cat# 16045-1) for three days, after which metastatic lung nodules were counted. The normalized number of lung metastases was calculated by dividing the number of lung metastases by the time between inoculation and termination of that mouse.

### Cell staining and flow cytometry

Spleens and peripheral blood were collected, homogenized, then red blood cells were lysed with Ammonium-Chloride-Potassium (ACK) lysis buffer for 2–3 min. All reagents were from Sigma-Aldrich. Cells were then stained with CD45 Pacific Blue, CD3 PE-Cy7, CD4 BV510, CD8 FITC, CD44 APC, CD62L APC-Fire750, CD27 Alexa700, CD19 PE-Dazzle594, and CD20 PercpCy5.5 antibodies (all from BioLegend) at 4°C for 30 min, followed by washing and resuspending in FACS buffer. Samples were collected and run on a Gallios (BD Biosciences) flow cytometer and analyzed with FlowJo 10 software.

### RNA extraction

For extracting RNA from PBMCs (to be used in TCR repertoire analysis), blood was collected on day 5 (before any radiation or immunotherapy) for all groups and 7-9 days after the last radiation fraction or anti-CTLA-4 for treatment groups. According to the manufacturer’s protocol, procedures were followed by sample filtration and RNA extraction with an RNeasy Mini Kit (Cat. #74136) from Qiagen (Hilden, Germany).

### Bioinformatics analysis for TCR sequencing

The raw TCR sequencing data were processed using MiXCR (19) (version 3.0.13) with default parameters. In brief, the raw reads were aligned to the Mus Musculus reference T-cell receptor genes based on the ImMunoGeneTics database (IMGT) (20). Then, the aligned reads were assembled to construct the Complementarity-determining region 3 (CDR3). Finally, MiXCR generated the clonotypes of each sample, where a unique clonotype was defined by its unique CDR3 amino acid sequence and V-J segments genes.

Further bioinformatics analysis and data visualization was performed using the Immunarch package [http://doi.org/10.5281/zenodo.3367200] in R (version 4.0.1). The clonality was defined as the Pielou’s evenness index, which was calculated using the formula: frequency/proportion of clone for a sample with a total of unique CDR3 sequences. The diversity of the repertories was measured using the Inverse Simpson index. The circlize package (21) was used to generate the circos plot of each sample regarding V-J usage.

Statistical analysis was performed using R (version 4.0.1). The comparisons of clonality and diversity among the five treatment groups were analyzed using the Kruskal–Wallis H test, followed by the *post hoc* Dunn’s test for pairwise comparisons. Statistical significance was considered at P-value < 0.05.

### Statistical analysis

GraphPad Prism 8.0 software was used to evaluate changes in tumor growth, survival, and flow cytometric findings. Tumor growth curves were compared using the 2-way analysis of variance. Mouse survival rates were analyzed with the Kaplan-Meier method and compared with log-rank tests. The number of spontaneous lung metastases was compared using 2-tailed t-tests. Differences between conditions were deemed significant at p < 0.05.

## Results

### Pulsed radiation results in better survival and lower lung metastases

**Figure 2:**
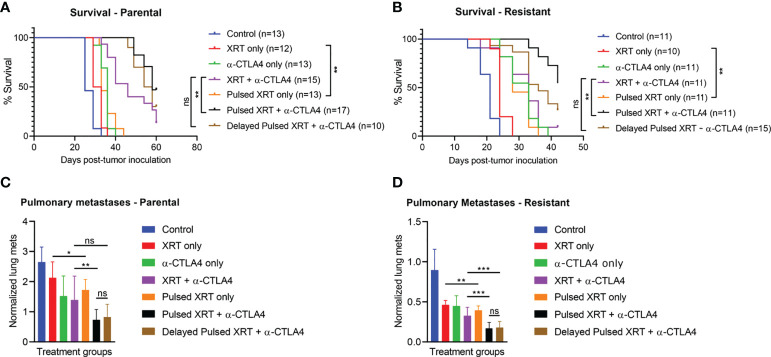
The combination of Pulsed XRT and anti-CTLA-4 treatment prolonged survival in both 344SQ-Parental and 344SQ-Resistant murine models. Survival curves of various groups in the 344SQ-Parental **(A)** and 344SQ-Resistant **(B)** models respectivley. **(C)** Normalized lung metastasis counts in 344SQ-Parental. **(D)** Noramlized lung metastasis counts in 344SQ-Resistant. *p < 0.05, **p < 0.01, ***p < 0.001.

In the parental cell line, Pulsed XRT with anti-CTLA-4 had higher survival compared to the XRT + anti-CTLA-4 group (p < 0.01) (**Figure 2A**). The survival benefit of the Pulsed XRT + anti-CTLA-4 group over XRT + anti-CTLA-4 was also observed in the resistant cell line (p < 0.01) (**Figure 2B**). However, there was no significant survival benefit with delayed Pulsed radiation and anti-CTLA-4.

When the Pulsed XRT and the XRT only groups were evaluated without immunotherapy, survival benefit was still statistically significant in favor of Pulsed XRT (p < 0.01, for both parental and resistant cell lines).

Pulsed XRT with anti-CTLA-4 group also had significantly lower lung metastases than XRT + anti-CTLA-4 group (p = 0.0051 for parental model; p = 0.0007 for resistant model) (**Figures 2C, D**).

### Pulsed radiation impairs primary tumor growth and controls high tumor burden

**Figure 3:**
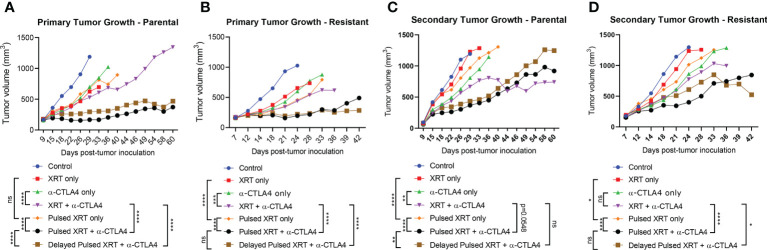
Pulsed radiation with anti-CTLA-4 checkpoint inhibitor generated a reverse abscopal effecton primary tumors of both 344SQ-Parental and 344SQ-Resistant models. **(A)** Primary tumor growth curves in the 344SQ-Parental model. **(B)** Primary tumor growth curves in the 344SQ-Resistant model. **(C)** Secondary tumor growth curves in the 344SQ-Parental model. **(D)** Secondary tumor growth in the 344SQ-Resisatnt model. Growth lines were compared between groups using two-way ANOVA and significance was set at p < 0.05. *p < 0.05, **p < 0.01, ***p<0.001, ****p < 0.0001.

Combining anti-CTLA-4 with radiation led to partial responses in the primary tumor. Pulsed radiation in combination with anti-CTLA-4 delayed primary tumor growth to a greater extent than one cycle of XRT + anti-CTLA-4 for both parental and resistant cell lines (Parental: p < 0.0001, Resistant: p < 0.0001) (**Figures 3A, B**). However, the advantage of Pulsed radiation + anti-CTLA-4 over one cycle of XRT + anti-CTLA-4 group on the secondary tumor growth was not as evident as the primary tumor (Parental: p = 0.0548, Resistant: p < 0.0001) (**Figures 3C, D**).

### Pulsed radiation expands memory T cells

**Figure 4:**
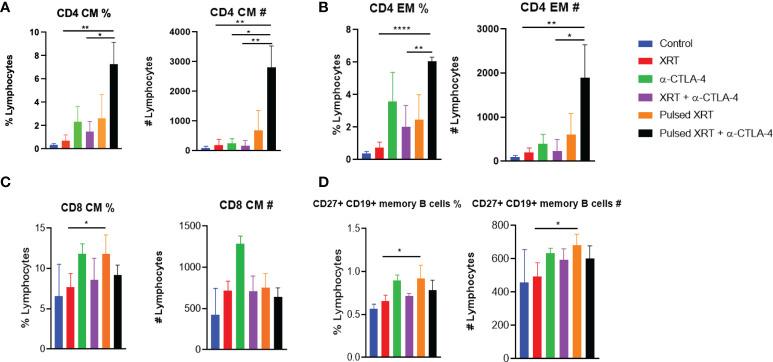
Flow cytometry analysis of lymphocytes for immune memory in the blood and spleen of treated mice on day 26. **(A)** CD4^+^ Central memory in blood as percentage and cell count. **(B)** CD4^+^ Effector memory in blood as percentage and cell count. **(C)** CD8^+^ central memory in spleen as percentage and cell count. **(D)** CD27^+^ CD19^+^ memory B cells in spleen as percentage and cell count. %, percentage; #, cell number; CM, Central memory; EM, Effector memory. Statistical signficance was determined between two groups using Student’s t-test. *p < 0.05, **p < 0.01, ****p < 0.0001.

We found that long-term memory was established in mice treated with Pulsed XRT + anti-CTLA-4. The pulsed radiation expanded tumor-specific CD4^+^ T cells and increased central as well as effector memory. Specifically, in the blood, the proportions of CD4^+^ cells increased with Pulsed XRT + anti-CTLA-4 treated mice relative to other groups (**Figures 4A, B**). When CD8^+^ cells and memory B cells were evaluated with flow cytometry, the only significant difference observed was between the Pulsed XRT and the XRT only groups. Adding a course of radiation (Pulsed XRT) increased the proportions of splenic CD8^+^ T cells and memory B cells (**Figures 4C, D**).

### Pulsed radiation expands T cell repertoire

**Figure 5:**
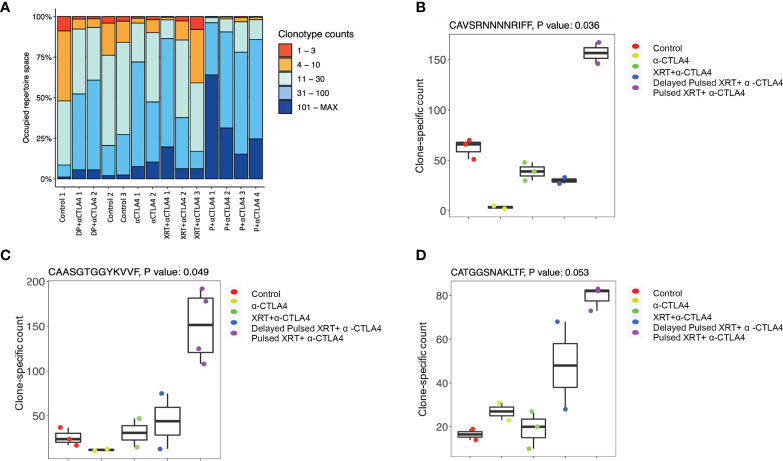
TCR repretoire analysis of different experimental groups. **(A)** Proportion of clonotypes with specific counts. The proportion of clonotypes with specific counts was higher in the pulsed XRT and anti-CTLA-4 group. DP, Delayed Pulsed XRT; P, Pulsed XRT. **(B–D)** Specific clones (CAVSRNNNNRIFF, p = 0.036; CAASGTGGYKVVF, p = 0.049; CATGGSNAKLTF, p = 0.053) in the T-cell receptor repertoire that were significantly high in the Pulsed XRT + α-CTLA-4 group.

Finally, because T cell-mediated control of tumors is associated with changes in the TCR repertoire, we analyzed the TCR-a and TCR-b repertoires from the blood to evaluate how specific changes in the T cell receptors might occur after Pulsed radiation. Our analyses focused on the complementarity-determining region 3 (CDR3) sequence of each TCR-a and TCR-b subunit and its component VDJ segment for TCR-b and VJ for TCR-a. Occupied repertoire space was computed for control (n = 3), anti-CTLA-4 only (n = 2), XRT + anti-CTLA-4 (n = 3), Pulsed XRT + anti-CTLA-4 (n = 4), and delayed Pulsed XRT + anti-CTLA-4 (n = 2) groups (**Figure 5A**). Although it was not statistically significant, the proportion of clonotypes with specific counts was higher in the Pulsed XRT and anti-CTLA-4 group. Some specific clones in the TCR repertoire were also identified that might contribute to the increased antitumor response with Pulsed radiation. In this context, CAVSRNNNNRIFF (p = 0.036), CAASGTGGYKVVF (p = 0.049), and CATGGSNAKLTF (p = 0.053) clones were relatively high in the Pulsed radiation + anti-CTLA-4 group compared to control and other treatment groups (**Figures 5B–D**).

## Discussion

The majority of cancer patients are diagnosed with a progressive disease that requires more than local radiation or surgical resection. Although radiation treatment can elicit an abscopal response, it is rare when radiation is used as a monotherapy (22, 23). Even with combinatorial chemoradiation, long-term survival remains low for most tumor types. Immunotherapeutic targets such as CTLA-4 and PD1 have proven efficacious in clinical trials (24–35). Despite their significant potential, the majority of patients are resistant to immunotherapy. Preclinical evidence suggests radiation and immunotherapies are synergistic in treating non-small cell lung cancer and other solid tumors (36, 37). As a result, a rapidly growing body of research is looking at combinatorial therapies to enhance the effects of immunotherapy in the clinic, particularly in patients who prove resistant to monotherapy (11, 38–43).

Our current study explains how the efficacy of anti-CTLA-4 might be increased with Pulsed XRT in order to control tumor burden and generate immune memory. First, we developed parental and checkpoint-resistant murine models. Then, we demonstrated that cycles of radiation (instead of single course therapy) drove a “reverse abscopal effect” at primary sites and expanded memory T cells, resulting in better survival. The addition of Pulsed XRT resulted in enhanced effector memory T cells and improved tumor control.

It is unclear whether an additional radiation cycle may promote or harm the continuing immune response. Pulsed XRT improved survival *via* durable tumor-specific memory, and the antitumor effects were largely dependent on CD4^+^ T cells. Timmerman et al. also evaluated an ablative dosing scheme called ‘personalized ultra-fractionated stereotactic adaptive radiotherapy’ (PULSAR). They showed the efficacy of ablative radiation when it was given in pulses of 10 days apart (16). They demonstrated that CD8 depleting antibodies abolished tumor control, but there was no information regarding CD4^+^ T cells and their function while building immune response in PULSAR. In our experiments, there was an evident increase of both central and effector CD4^+^ T cell memory upon Pulsed radiation treatment, while there were no apparent shifts in the CD8^+^ population. It is important to note other experimental variables for these two studies. First, while one-tumor model was used in PULSAR, our experimental design included bilaterally established tumors (two-tumor model) to generate higher burden. Also, we directed the Pulsed cycle of radiation to the secondary tumors, which may explain the tumor-specific T cell distribution in the peripheral blood. In addition, different checkpoint inhibitors were utilized in PULSAR vs our current study.

Although using Pulsed radiation with anti-CTLA-4 was efficient to control both treatment sites, in our observations the tumor response in the primary tumor was more evident than the secondary tumor. In contrary to conventional abscopal settings, we prospectively irradiated the secondary site, which led to an amplified effect back on the primary tumor. However, irradiating the secondary sites in the 344SQ-Parental model compromised the antitumor efficacy at these secondary locations that we otherwise observe with abscopal treatments (XRT + anti-CTLA-4 group). The relatively weaker results in secondary tumors of pulsed groups may be associated with the negative effects of high dose radiation on newly recruited T cells to the secondary sites. On the other hand, in the 344SQ-Resistant model, secondary tumors showed significant response with Pulsed XRT + anti-CTLA-4 treatment. This is due to the higher sensitivity of 344SQR (aggressively-dividing model) to high dose XRT, despite the ablative effect on recruited T cells.

Our previous study hypothesized that several rounds of radiation (Pulsed-XRT) would likely provide more significant clinical benefits to patients with metastatic disease (17). This hypothesis was based on the concept of conventional vaccines that benefit from “booster cycles” to generate long-term memory. Therefore, in cancer settings, tumor-associated antigens may be generated with each radiation cycle to enhance cellular and humoral memory. Moreoever, we have previously developed an *in-vivo* model similar to our current experimental design and evaluated the T-cell stimulation and effector functions through cytokine analysis. In this context, IL-1a and IL-1b proinflammatory cytokines were significantly elevated (p = 0.04) with Pulsed XRT compared to XRT (17). IL-12 (p70) cytokine and IFN-g were also upregulated, shifting the balance towards Th1 antitumor responses. In addition, TNF-α cytokine was elevated with Pulsed XRT, which is previously shown by others to mediate abscopal responses (44) and favor M1 macrophage polarization (45). One of the most critical functions of Pulsed radiation could be attributed to its ability to release different neoantigens after irradiating different metastatic sites. Therefore, every pulse may contribute not only to TCR repertoire clonality but also to overall diversity. Our study shows that some specific clones in Pulsed radiation + anti-CTLA-4 group increased significantly, which may contribute to the antitumor response. In addition, clonality was found to be higher with Pulsed radiation, although it did not reach significance. A limitation of this study could be the fact that phenotyping and TCR repertoire results were dependent on systemic blood-draw analysis only. Correlative assessment of tumor tissues may strengthen our hypothesis and complement the blood data.

Further research is warranted to understand how various treatment parameters for Pulsed XRT may influence outcomes and modulate the immune system upon repeated antigen exposure. For example, we recommend that pulse radiation be delivered in hypofractionated fashion (i.e. SBRT), this is because prolonged fractionation will reduce/eliminate immune cells and diminish the immune memory observed herein. Another parameter is the type of beam used. It would be interesting to test pulsed XRT in context of protons instead of photons in the near future in combination with different checkpoint inhibitors, while keeping toxicity aspects in mind.

In conclusion, Pulsed XRT is a safe and feasible way to improve tumor control, acting as a vaccine to repeatedly stimulate and expand the immune response. Patients with polymetastatic disease may be the most beneficiary cohort in the clinic from Pulsed XRT treatment, due to its contribution to systemic immune memory and ease of application with existing technologies.

## Data availability statement

The original contributions presented in the study are included in the article/supplementary material. Further inquiries can be directed to the corresponding author.

## Ethics statement

The animal study was reviewed and approved by The University of Texas MD Anderson Cancer Center IACUC committee.

## Author contributions

Writing the manuscript: DS, HB, EH. Conducting experiments and data acquisition: DS, HB, KH, MW. Interpretation of data and analysis: QW, JW, CA, DS, HB, EH. Reviewing and editing the manuscript: DS, HB, KH, YH, QW, CA, N-PO, EH, MW, FM, JW, MC, JWW. Supervision: JWW. All authors contributed to the article and approved the submitted version.

## Funding

This work was supported in part by Cancer Center Support (Core) Grant P30 CA016672 from the National Cancer Institute, National Institutes of Health, to The University of Texas MD Anderson Cancer Center.

## Conflict of interest

JWW reports research support from GlaxoSmithKline, Bristol Meyers Squibb, Merck, Nanobiotix, RefleXion, Alkermes, Artidis, Mavu Pharma, Takeda, Varian, Checkmate Pharmaceuticals and HotSpot Therapeutics. JWW serves/served on the scientific advisory board for Legion Healthcare Partners, RefleXion Medical, MolecularMatch, Merck, AstraZeneca, Aileron Therapeutics, OncoResponse, Checkmate Pharmaceuticals, Mavu Pharma, Alpine Immune Sciences, Ventana Medical Systems, Nanobiotix, China Medical Tribune, GI Innovation, Genentech and Nanorobotix. JWW serves as consultant for Lifescience Dynamics Limited. JWW has/had Speaking Engagements for Ventana Medical Systems, US Oncology, Alkermes, Boehringer Ingelheim, Accuray and RSS. JWW holds/held stock or ownership in Alpine Immune Sciences, Checkmate Pharmaceuticals, Healios, Mavu Pharma, Legion Healthcare Partners, MolecularMatch, Nanorobotix, OncoResponse, and RefleXion. JWW has accepted honoraria in the form of travel costs from Nanobiotix, RefleXion, Varian, Shandong University, The Korea Society of Radiology, Aileron Therapeutics and Ventana. JWW has the following patents: MP470 (amuvatinib), MRX34 regulation of PDL1, XRT technique to overcome immune resistance, Radiotherapies and uses thereof. MD Anderson Cancer Center has a trademark for RadScopal™.

HB has the following patent: Radiotherapies and uses thereof.

The authors declare that the research was conducted in the absence of any commercial or financial relationships that could be construed as a potential conflict of interest.

## Publisher’s note

All claims expressed in this article are solely those of the authors and do not necessarily represent those of their affiliated organizations, or those of the publisher, the editors and the reviewers. Any product that may be evaluated in this article, or claim that may be made by its manufacturer, is not guaranteed or endorsed by the publisher.
